# Novel Co_3_O_4_ Nanoparticles/Nitrogen-Doped Carbon Composites with Extraordinary Catalytic Activity for Oxygen Evolution Reaction (OER)

**DOI:** 10.1007/s40820-017-0170-4

**Published:** 2017-11-14

**Authors:** Xiaobing Yang, Juan Chen, Yuqing Chen, Pingjing Feng, Huixian Lai, Jintang Li, Xuetao Luo

**Affiliations:** 1 College of Ecology and Resource Engineering, Wuyi University, Wuyishan, 354300 Fujian People’s Republic of China; 2 Department of Pharmacy, Zhongshan Hospital, Xiamen University, Xiamen, 361004 Fujian People’s Republic of China; 3 Fujian Provincial Key Laboratory of Eco-Industrial Green Technology, Wuyi University, Wuyishan, 354300 Fujian People’s Republic of China; 4 Fujian Key Laboratory of Advanced Materials, College of Materials, Xiamen University, Xiamen, 361005 Fujian People’s Republic of China

**Keywords:** Co_3_O_4_ nanoparticles, Nitrogen-doped carbon, ZIF-67, Catalytic, Oxygen evolution reaction (OER)

## Abstract

Herein, Co_3_O_4_ nanoparticles/nitrogen-doped carbon (Co_3_O_4_/NPC) composites with different structures were prepared via a facile method. Structure control was achieved by the rational morphology design of ZIF-67 precursors, which were then pyrolyzed in air to obtain Co_3_O_4_/NPC composites. When applied as catalysts for the oxygen evolution reaction (OER), the M-Co_3_O_4_/NPC composites derived from the flower-like ZIF-67 showed superior catalytic activities than those derived from the rhombic dodecahedron and hollow spherical ZIF-67. The former M-Co_3_O_4_/NPC composite displayed a small over-potential of 0.3 V, low onset potential of 1.41 V, small Tafel slope of 83 mV dec^−1^, and a desirable stability. (94.7% OER activity was retained after 10 h.) The excellent performance of the flower-like M-Co_3_O_4_/NPC composite in the OER was attributed to its favorable structure.

**Electronic supplementary material:**

The online version of this article (10.1007/s40820-017-0170-4) contains supplementary material, which is available to authorized users.

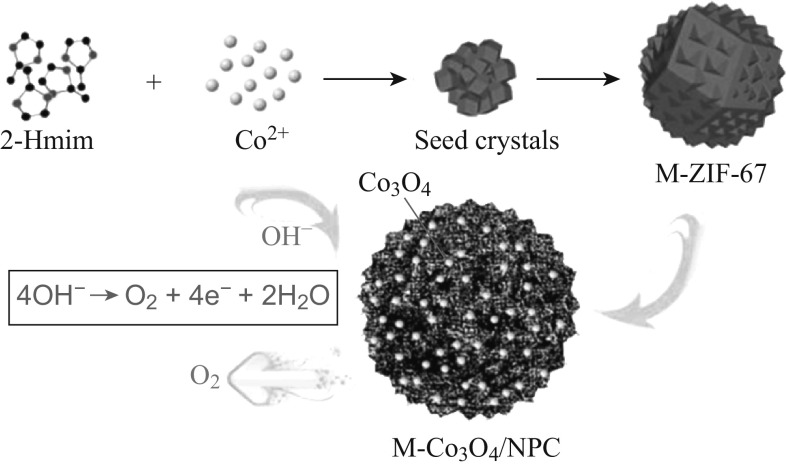

## Highlights

- Co_3_O_4_ nanoparticles/nitrogen-doped carbon (Co_3_O_4_/NPC) composites were successfully fabricated from zeolitic imidazolate framework 67 (ZIF-67), and the composite structure could be well controlled by adjusting the structure of ZIF-67.
- M-Co_3_O_4_/NPC composites derived from flower-like ZIF-67 showed the highest activities for the oxygen evolution reaction (OER).

## Introduction

Depletion of fossil fuels and the rapidly growing energy demands have necessitated the development of sustainable energy conversion and storage systems such as metal–air batteries, water splitting devices, and fuel cells [1–4]. The development of durable, highly efficient, low-cost, and eco-friendly electrocatalysts for the oxygen evolution reaction (OER) is crucial for the commercial application of these renewable energy technologies [5, 6]. To date, precious metal-based materials, such as RuO_2_ and IrO_2_, have been considered as the most optimal catalysts for OER owing to their lowest over-potentials at practical current densities [7]. However, their commercial applications have been severely impeded because of their poor stability, prohibitive cost, and low selectivity [8].

Recently, significant efforts have been made to explore transition metal-based electrocatalysts for the OER because of their low cost, abundant reserves, environmental benignity, and resistance to corrosion in alkaline solutions [9–12]. Among them, Co-based catalysts have emerged as promising alternatives for precious metal-based catalysts [13–16]. The electrocatalytic activity for OER is closely related to the active sites and electronic conductivity of the catalysts. Previous research has demonstrated that active sites can be engineered by modulating the particle size, pore structure [17, 18], and the crystallinity [19, 20] of Co_3_O_4_. Furthermore, coupling with carbon effectively improves the electronic conductivity of the catalysts [21–23]. Nevertheless, carbon itself as a catalyst displays relatively low catalytic OER activity. Recent studies have shown that doping with either nitrogen or transition metals into carbon nanostructure can efficiently promote its catalytic performance [23–26]. The template method has proven to be an effective protocol for obtaining nitrogen-doped Co_3_O_4_/C composites. In this method, various organic hybrids, which contain both the transition metal and nitrogen, are used as precursors such as melamine [27], porphyrin [28], polyaniline [29, 30], and salen [31]. However, it is hard to control the size, structure, and morphology of these organic hybrids in an exact manner; therefore, deficiencies and non-uniform distributions of active sites are prevalent, which are also crucial for electrocatalytic activity.

Metal organic frameworks (MOFs) have attracted a significant attention as materials for the preparation of non-precious metal electrocatalysts because of their inherent advantages such as a controllable porous structure, innate doping with heteroatoms, and an ultrahigh surface area [32, 33]. Zeolitic imidazolate frameworks (ZIFs) have proven to be promising as pyrolytic precursors for various porous metal oxides/doped carbon composites [34–36]. Via direct pyrolysis, carbon layers with a porous structure can be formed in situ with metal nanoparticles encapsulated homogeneously, and sufficient contacts can be formed between the metal nanoparticles and the carbon matrix. Notably, a highly ordered three-dimensional structure promotes the structural stability of MOFs against pyrolysis, and the remarkable surface-to-volume ratio of MOFs can effectively promote the electrochemical catalytic reactions.

Among the variety of MOF materials available, ZIF-67 is one of the most widely investigated ones because of its high concentration of active cobalt sites as well as a facile synthetic method. Herein we have proposed a facile method to prepare Co_3_O_4_/NPC composites with different morphologies derived from ZIF-67. By slightly modulating the synthetic route of the ZIF-67 precursors, it was possible to control the morphology of the product. Thus, in addition to the typical rhombic dodecahedron morphology, novel flower-like ZIF-67 and hollow spherical ZIF-67 were fabricated. These ZIF-67 precursors were then pyrolyzed to obtain the Co_3_O_4_/NPC composites of different structures, named T-Co_3_O_4_/NPC, M-Co_3_O_4_/NPC, and H-Co_3_O_4_/NPC, respectively. The electrocatalytic activities for OER of the three composites were then investigated to determine the most favorable morphology for the highest electrocatalytic performance for the OER.

## Experimental

### Chemicals

Cobalt nitrate hexahydrate (Co(NO_3_)_2_·6H_2_O, > 99.8%) was purchased from Shanghai Titanchem Co. Ltd., and methanol (CH_3_OH, > 99.5%), cobalt sulfate heptahydrate (CoSO_4_·7H_2_O, > 99.8%), 2-methylimidazole (C_4_H_6_N_2_, 99%), and polyvinylpyrrolidone ((C_6_H_9_NO)_n_) were obtained from Sinopharm Chemical Reagent Co. Ltd. All reagents were used as received without further purification.

### Preparation of ZIF-67 Precursors

T-ZIF-67 was synthesized according to a previously reported method [37]. In a typical procedure, solutions of Co(NO_3_)_2_·6H_2_O (5.82 g) in methanol (400 mL) (solution A) and 2-methylimidazole (6.48 g) in methanol (400 mL) (solution B) were prepared. Solution B was gradually added into solution A with continuous stirring. After standing for a while, layers were observed and the supernatant was eliminated. The solution was then centrifuged and washed with methanol for 3–5 times to remove the excess Co^2+^. T-ZIF-67 was finally acquired as a purple solid after drying at 60 °C for 3 h.

The synthetic route to M-ZIF-67 was almost the same as that of T-ZIF-67, except that Co(NO_3_)_2_·6H_2_O was replaced by CoSO_4_·7H_2_O (5.62 g). During the synthesis of H-ZIF-67, 1.00 g PVP was added to the methanol solution of 2-methylimidazole as a morphology modifier, and other steps were the same as that for the synthesis of M-ZIF-67.

### Preparation of Co_3_O_4_/NPC Composites

The as-prepared M-ZIF-67, H-ZIF-67, and T-ZIF-67 materials were first ground into powders. They were then individually heated to 550 °C in air at a heating rate of 5 °C min^−1^. After keeping at 550 °C for 5 h, the powdered materials were cooled down to room temperature at a cooling rate of 5 °C min^−1^ and black M-Co_3_O_4_/NPC, H-Co_3_O_4_/NPC, and T-Co_3_O_4_/NPC powders were obtained, respectively.

### Characterization

Powder X-ray diffraction (PXRD) analysis of the materials was performed on a Bruker-AXS D8 Advance X-ray diffractometer with Cu Kα radiation (*λ* = 0.15406 nm). The morphologies and elemental mappings of the samples were obtained from a Hitachi SU70 field-emission scanning electron microscopy (SEM) instrument at 10 kV and 20 kV. The high-resolution transmission electron microscopy (HRTEM) characterization was carried out on a Tecnai F30 microscope at an accelerating voltage of 300 kV. Elemental analysis was performed on a Vario EL III elemental analyzer. The specific surface area and pore size distribution were determined by the Brunauer–Emmett–Teller (BET) method conducted by the TriStar II 3020 surface area and porosity analyzer. Thermogravimetric analysis (TGA) of the samples was carried out on a SDTQ600 thermoanalyzer in air. X-ray photoelectron spectroscopy (XPS) was performed on a Thermo Scientific ESCALAB 250Xi with Al Kα radiation (*hν* = 1486.8 eV).

### Electrochemical Measurements

Cyclic voltammetry (CV) and linear sweep voltammetry (LSV) measurements were taken on an Autolab PGSTAT302N electrochemical workstation (NOVA 1.9). The evaluation of the catalytic activity for the OER was conducted at room temperature in a conventional three-electrode system. Co_3_O_4_/NPC composites were used as the working electrode, a platinum foil acted as a counter electrode, and a reversible hydrogen electrode (RHE) was employed as the reference electrode. To prepare the working electrode, 5 mg of the active material was dispersed in a mixture of 0.95 mL ethanol and 0.05 mL 5 wt% Nafion solution with sonication for 60 min. Next, the catalyst (20 μL) was pipetted out and dropped onto a glassy carbon electrode with a diameter of 5 mm. It was then fully dried at room temperature for 12 h before measurements (loading ~0.510 mg cm^−2^).

## Results and Discussion

Figure S1 shows the PXRD patterns of M-ZIF-67, H-ZIF-67, and T-ZIF-67. Apparently, these three materials exhibit the same XRD pattern with principal diffraction peaks at 7.39°, 10.43°, 12.73°, and 18.07°, which are exactly matched with the simulated ZIF-67 pattern. This suggests that the three ZIFs have the same composition. This result was also supported by their FTIR spectra (Fig. S2). The diffraction peaks of T-ZIF-67 were much higher than those of M-ZIF-67 and H-ZIF-67, implying a higher crystallinity of T-ZIF-67 in comparison with the other two ZIF-67 precursors.

**Fig. 1:**
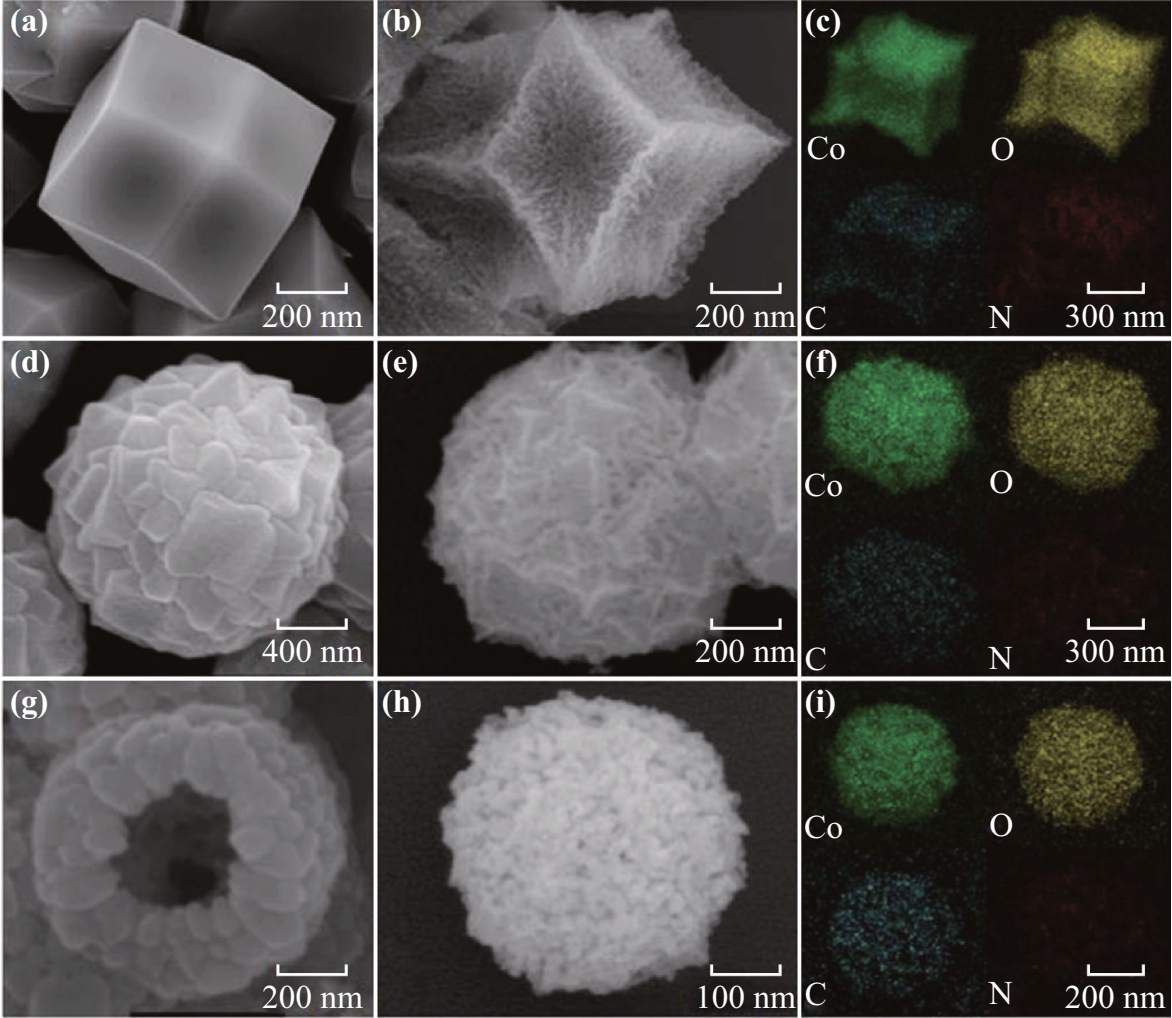
SEM images of **a** T-ZIF-67, **b** T-Co_3_O_4_/NPC**, d** M-ZIF-67, **e** M-Co_3_O_4_/NPC**, g** H-ZIF-67, and **h** H-Co_3_O_4_/NPC. Elemental mapping of **c** T-Co_3_O_4_/NPC, **f** M-Co_3_O_4_/NPC, and **i** H-Co_3_O_4_/NPC

**Fig. 2:**
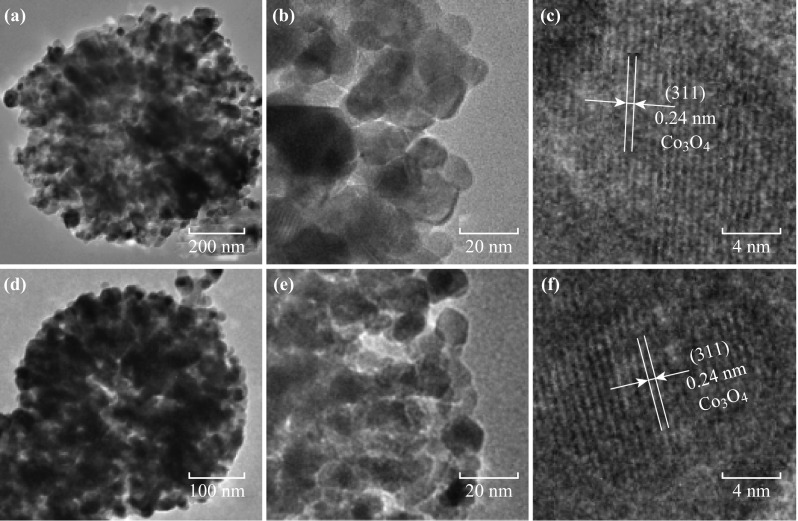
TEM images of **a–c** M-Co_3_O_4_/NPC and **d–f** H-Co_3_O_4_/NPC composites

The morphologies of the ZIF-67 precursors and the as-prepared Co_3_O_4_/NPC composites were studied by SEM. T-ZIF-67 showed a rhombic dodecahedron morphology with particle sizes of ~1 μm, which is the typical morphology of ZIF-67 (Fig. 1a). On the other hand, the morphology of M-ZIF-67 was flower-like with particles of size ~1.6 μm (Fig. 1b) and that of H-ZIF-67 was hollow spherical with a diameter of ~800 nm and shell thickness ~200 nm (Fig. 1g, h). After pyrolysis at 550 °C for 5 h under air, all three ZIF-67-derived composites inherited the morphologies of their precursors without either particle agglomeration (Fig. S3) or structural collapse, indicating a high structural stability of the obtained Co_3_O_4_/NPC composites. Specifically, the surfaces of T-ZIF-67 shrunk into a rhombic dodecahedron center with Co_3_O_4_ nanoparticles uniformly embedded in the carbon scaffold (Fig. 1b). M-ZIF-67 and T-ZIF-67 underwent similar changes in morphology to yield M-Co_3_O_4_/NPC (Fig. 1e) and H-Co_3_O_4_/NPC (Fig. 1h), respectively. To determine the elemental composition of the composites, elemental mapping analysis was conducted. As shown in Fig. 1c, f, i and Table S1, all three composites were mainly comprised of cobalt and oxygen, with trace amounts of carbon and nitrogen. This implied that the pyrolysis of ZIF-67 yields a nitrogen-doped carbon scaffold encapsulated in situ with Co_3_O_4_ nanoparticles. Further detailed investigations were performed by using HRTEM (Fig. 2). The Co_3_O_4_ nanoparticles adsorbed on the M-Co_3_O_4_/NPC and H-Co_3_O_4_/NPC composites were of similar sizes at ~12 nm. The lattice distance in the related high-resolution TEM images matched the (311) interplanar distance of the Co_3_O_4_ nanoparticles.

**Fig. 3:**
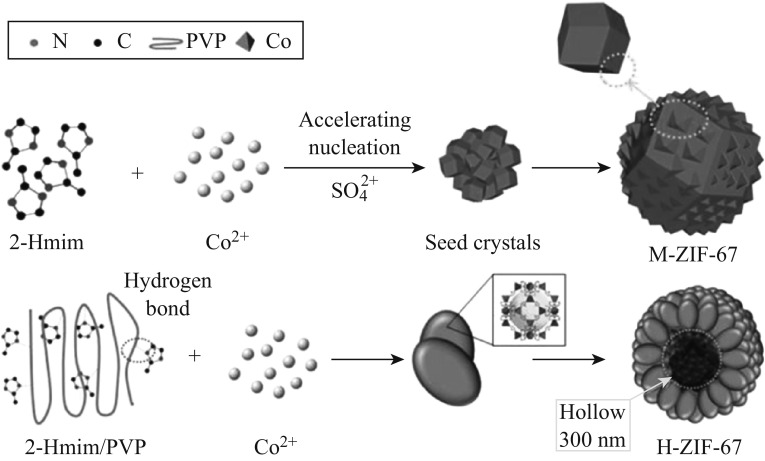
Schematic diagram of the preparation of **a** M-ZIF-67 and **b** H-ZIF-67

To clearly illustrate the process of morphology control, the schematic diagrams of the synthetic procedure are presented in Fig. 3. In the traditional synthetic method of ZIF-67, Co(NO_3_)_2_·6H_2_O has been used as the metal source. In this work, we used CoSO_4_·7H_2_O as the metal source instead. The introduction of SO_4_^2+^ species accelerated the nucleation of ZIF-67, leading to multiple polyhedrons being embedded mutually, until finally flower-like ZIF-67 particles had formed. As for H-ZIF-67, PVP was employed as a template. As shown in Fig. 3, 2-methylimidazole combined with the PVP molecular chain via hydrogen bonds when they were dissolved together in methanol. This interaction between the ligands and the template forced ZIF-67 to grow along the chain, resulting in flake-like ZIF-67, which then piled together to form a hollow sphere.

**Fig. 4:**
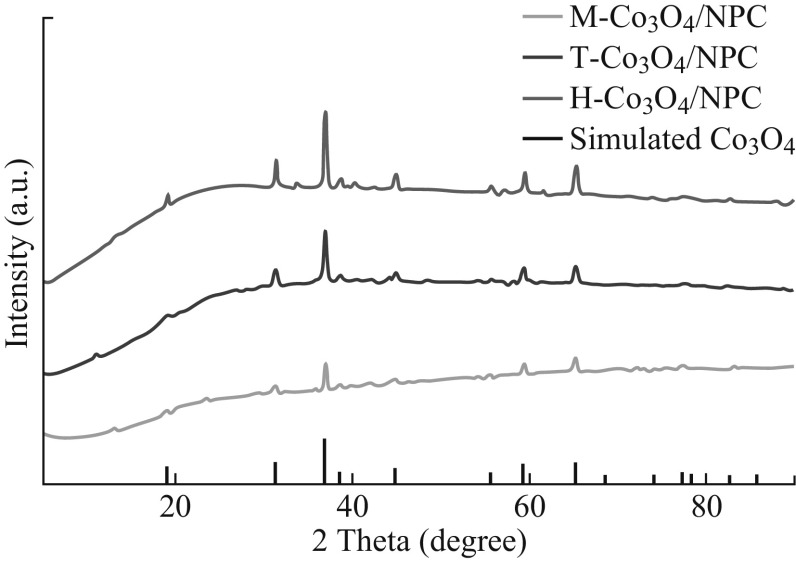
PXRD patterns of T-Co_3_O_4_/NPC, M-Co_3_O_4_/NPC, and H-Co_3_O_4_/NPC composites

The PXRD patterns of the T-Co_3_O_4_/NPC, M-Co_3_O_4_/NPC, and H-Co_3_O_4_/NPC composites were obtained to investigate their compositions. As shown in Fig. 4, apart from the differences in diffraction intensities, the XRD patterns of the three composites were the same. Peaks at 31.27°, 36.85°, 44.81°, 59.36°, and 65.24° could be indexed to the (220), (311), (400), (511), and (440) planes of spinel cobalt oxide (JCPDS No. 42-1467), respectively. Since intense diffractions imply higher degree of crystallinity, it was concluded that the structure of M-Co_3_O_4_/NPC was the least ordered.

**Fig. 5:**
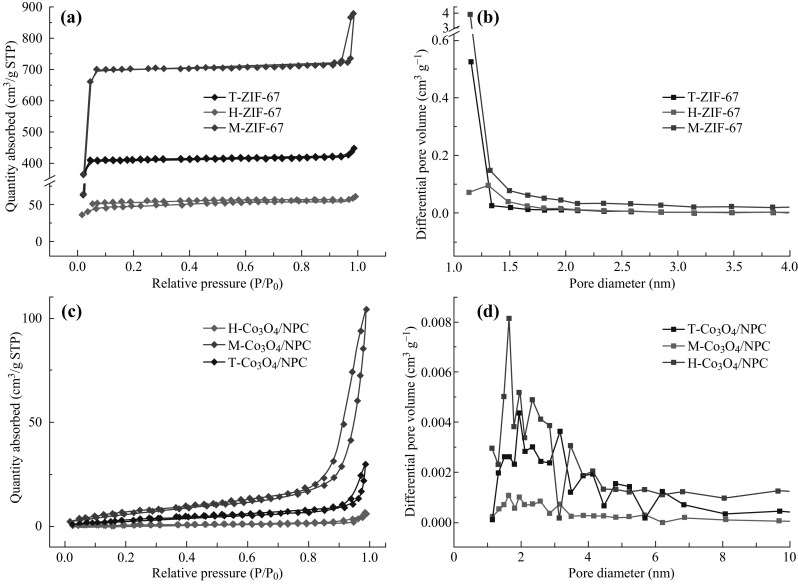
**a** Nitrogen absorption–desorption isotherms and **b** pore size distributions of M-ZIF-67, H-ZIF-67, and T-ZIF-67. **c** Nitrogen absorption–desorption isotherms and **d** pore size distributions of T-Co_3_O_4_/NPC, M-Co_3_O_4_/NPC, and H-Co_3_O_4_/NPC composites

To gain an in-depth understanding of the pore structure of the three composites, the nitrogen adsorption–desorption isotherms and the corresponding pore size distribution curves of ZIF-67 precursors and Co_3_O_4_/NPC composites were determined. As shown in Fig. 5a, the nitrogen adsorption–desorption isotherms of M-ZIF-67, H-ZIF-67, and T-ZIF-67 agreed with Langmuir I. In M-ZIF-67, the quantity of adsorbed N_2_ increased dramatically at a low relative pressure, indicating abundant micropores in the flower-like particles. Besides, at the tail of the isotherm (high relative pressure), the absorbance increased quickly, suggesting a large amount of mesopores. Similarly, T-ZIF-67 possessed numerous micropores with a relatively negligible number of mesopores. In contrast, H-ZIF-67 had much less of both micro- and mesopores. These differences are also evident in the pore size distribution curves (Fig. 5b). The BET surfaces of M-ZIF-67, H-ZIF-67, and T-ZIF-67 were determined to be 2375.343, 149.292, and 1187.203 m^2^ g^−1^, respectively. Accordingly, after pyrolysis, their BET surface areas were 25.869, 2.742, and 11.703 m^2^ g^−1^, respectively. Noticeably, the adsorption type changed from Langmuir I to Langmuir III after pyrolysis (Fig. 5c), and the pore sizes became larger and the distribution was more dispersive (Fig. 5d). On the basis of these results, it could be concluded that the pores in the Co_3_O_4_/NPC composites were mainly mesopores.

**Fig. 6:**
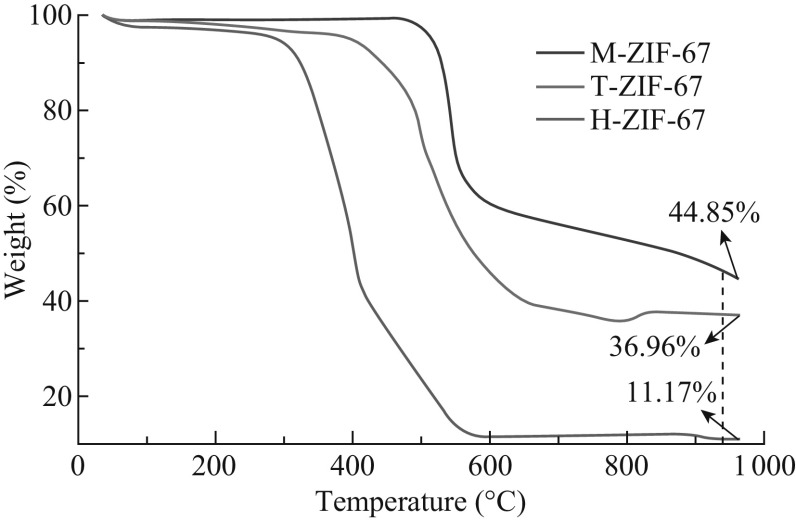
TGA curves of M-ZIF-67, H-ZIF-67, and T-ZIF-67

The thermal stabilities of the three composites were investigated by TGA under air atmosphere. As shown in Fig. 6, heavy mass losses for M-ZIF-67, H-ZIF-67, and T-ZIF-67 started at 550, 300, and 400 °C, respectively. When the temperature increased to 950 °C, the weights remained at 44.85%, 11.17%, and 36.96%, respectively. The dramatic weight loss was attributed to the combustion of the carbon species. It is noteworthy that both H-ZIF-67 and T-ZIF-67 went through a slight mass loss before decomposition, while M-ZIF-67 was stable below 500 °C. This phenomenon indicated that the thermal stability of M-ZIF-67 was much superior to that of H-ZIF-67 and T-ZIF-67.

**Fig. 7:**
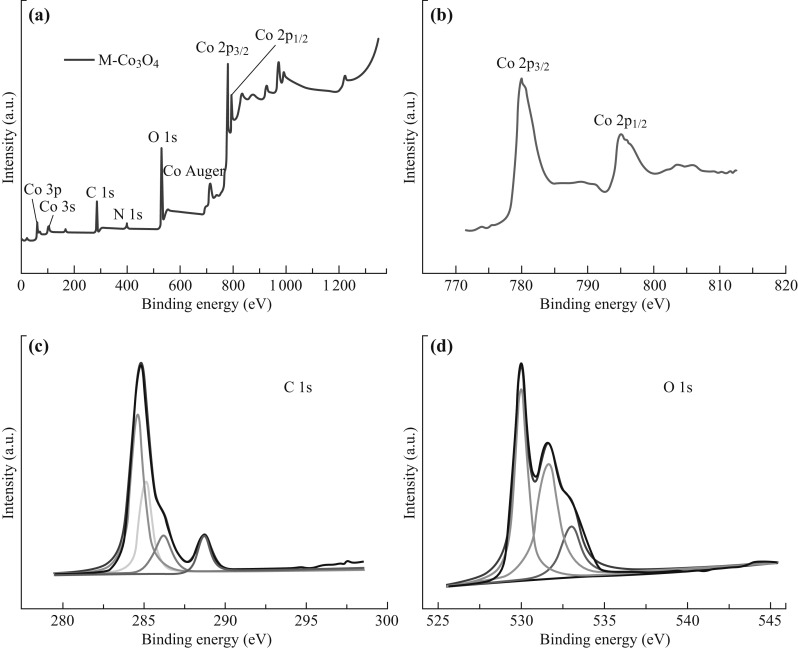
**a** Full XPS spectrum of M-Co_3_O_4_/NPC, deconvoluted spectra of **b** Co 2*p*, **c** C 1*s*, and **d** O 1*s*

Figure 7 shows the XPS results of the M-Co_3_O_4_/NPC catalyst. As shown in Fig. 7a, the full XPS spectra provided evidence for the presence of Co, O, and C. For the regional Co 2*p* spectrum, two major peaks at 780.0 and 795.0 eV were observed, which were correlated to the Co 2*p*_3/2_ and Co 2*p*_1/2_ spin–orbit peaks of Co_3_O_4_, respectively. In addition, two shakeup satellites, which were characteristic of Co_3_O_4_, were clearly observed at 789.9 and 804.3 eV [38]. The high-resolution spectrum of O 1*s* could be deconvoluted to three subpeaks (Fig. 7d). Peaks centered at 530.0 and 531.6 eV were assigned to the lattice oxygen (denoted as O_L_) in the Co_3_O_4_ phase and the O^2−^ ions in oxygen-deficient regions within the matrix of Co_3_O_4_ (denoted as O_D_), respectively. The peak at 533.0 eV was attributed to the absorbed oxygen species (O_A_). The percentage of O_D_ in the total oxygen content related to the defect sites was calculated from the spectrum as 41.5%. Such a high percentage of defect sites-related oxygen supported the high electrocatalytic performance of the M-Co_3_O_4_/NPC composite. The C 1*s* spectrum was deconvoluted into four subpeaks. The peak at 284.62 eV was attributed to the *sp* ^2^-hybridized graphite-like carbon (C–C *sp* ^2^), and the peak at 285.11 eV was correlated to both the *sp* ^3^-hybridized diamond-like carbon (C–C *sp* ^3^) and the *sp* ^2^-hybridized nitrogen-bonded carbon (C–N *sp* ^2^). The other two peaks centered at 286.19 and 288.70 eV were assigned to the carbon bonded with surface oxygen and nitrogen groups (C=O/C=N, O=C–O, and C–O/C–N), respectively [22, 39].

**Fig. 8:**
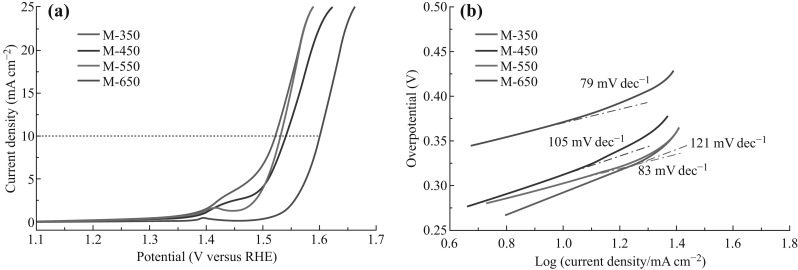
**a** LSV curves of M-350, M-450, M-550, and M-650 in O_2_-saturated 1.0 M KOH solution (scan rate 5 mV s^−1^), **b** Tafel plots of the synthesized catalysts

To determine the optimum pyrolysis temperature for OER, the flower-like ZIF-67 was pyrolyzed at different temperatures (350, 450, 550, and 650 °C). The electrochemical activities of M-350, M-450, M-550, and M-650 for OER were tested in O_2_-saturated 1.0 M KOH solution. The over-potential at a current density of 10 mA cm^−2^ is an important metric related to solar fuel synthesis. As shown in the LSV curves (Fig. 8a), M-350, M-450, and M-550 showed comparative catalytic activity, and the over-potentials at a current density of 10 mA cm^−2^ were 290, 310, and 302 mV, respectively. M-650 displayed a relatively poor catalytic activity with a high over-potential (~370 mV). However, the Tafel slopes revealed the opposite tendency. Tafel plots were established based on the LSV curves (Fig. 8b). The Tafel slope *b* is a parameter that describes the kinetics of the electrocatalyst for OER, which is determined by the Tafel equation:

1 $$\eta = a + b \log \left| J \right|,$$

where *η* refers to the over-potential, *b* represents the Tafel slope, and the current density is indicated by *J*. A smaller value of *b* implies a faster increase in the rate of the OER as applied to an increase in the potential. The Tafel slope values for M-550 and M-650 were 83 and 79 mA dec^−1^, much smaller than those of M-350 (~121 mA dec^−1^) and M-450 (~105 mA dec^−1^). In order to explain these results, the composition and structure analysis was performed by powder XRD. As shown in Fig. S4, the intensity of the diffraction peaks of Co_3_O_4_ increased with the pyrolysis temperature, indicating a highly disordered structure of M-350. As the TGA results (Fig. 6) revealed that there was no obvious weight loss from the M-ZIF-67 sample at 350 °C, it was reasonable to conclude that M-350 contained a high percentage of carbon. While a highly disordered structure efficiently improved the catalytic activity, the kinetics were compromised by the high carbon content. Remarkably, M-550 performed well in both metrics. Therefore, the optimum pyrolysis temperature was chosen as 550 °C.

**Fig. 9:**
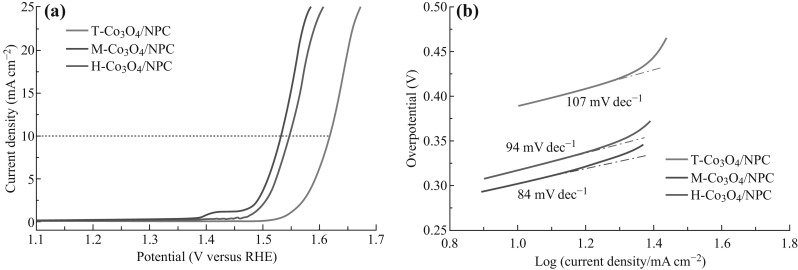
**a** LSV curves of the M-Co_3_O_4_/NPC, H-Co_3_O_4_/NPC, and T-Co_3_O_4_/NPC composites in O_2_-saturated 1.0 M KOH solution (scan rate 5 mV s^−1^), **b** Tafel plots of the prepared catalysts

Therefore, M-ZIF-67, H-ZIF-67, and T-ZIF-67 were pyrolyzed at 550 °C under air. As shown in the LSV curves (Fig. 9a), H-Co_3_O_4_/NPC and T-Co_3_O_4_/NPC exhibited relatively poor catalytic activity with onset potentials of 1.48 and 1.55 V, respectively, while M-Co_3_O_4_/NPC displayed a higher OER response with a low onset potential of 1.41 V. Among the three samples, M-Co_3_O_4_/NPC afforded a current density of 10 mA cm^−2^ at an over-potential of 302 mV, which was lower than those of H-Co_3_O_4_/NPC (~317 mV) and T-Co_3_O_4_/NPC (~388 mV), indicating that a flower-like morphology was more favorable for OER. The Tafel slope value for M-Co_3_O_4_/NPC was 84 mA dec^−1^, lower than those of H-Co_3_O_4_/NPC (94 mA dec^−1^) and T-Co_3_O_4_/NPC (107 mA dec^−1^) as well. These results suggested that the M-Co_3_O_4_/NPC composite derived from the flower-like ZIF-67 exhibited superior catalytic activity over T-Co_3_O_4_/NPC and H-Co_3_O_4_/NPC, which had been derived from rhombic dodecahedron and hollow spherical ZIF-67.

**Fig. 10:**
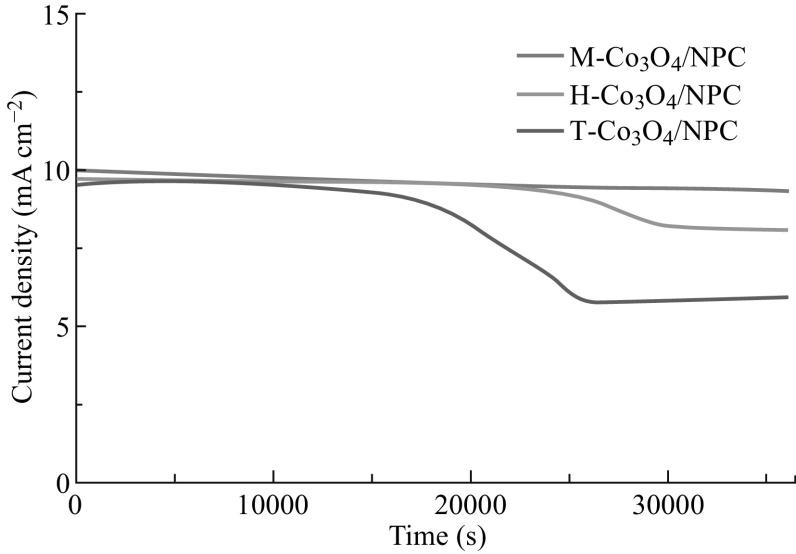
Chronoamperometric responses of the M-Co_3_O_4_/NPC, H-Co_3_O_4_/NPC, and T-Co_3_O_4_/NPC composites at constant potentials of 1.53, 1.62, and 1.55 V, respectively

Strong durability toward OER is of great significance for energy conversion and storage systems. The chronoamperometric responses of M-Co_3_O_4_/NPC, H-Co_3_O_4_/NPC, and T-Co_3_O_4_/NPC were determined at constant potentials of 1.53, 1.55, and 1.62 V, respectively. As shown in Fig. 10, M-Co_3_O_4_/NPC displayed superior stability in comparison with H-Co_3_O_4_/NPC and T-Co_3_O_4_/NPC, with only a slight anodic current attenuation of 5.3% within 10 h. This result was attributed to the excellent structural stability of the flower-like carbon scaffold, which was also evident by the TGA results. Notably, the M-Co_3_O_4_/NPC composite showed a better OER activity compared to not only most Co-based electrocatalysts, but also noble metal-based catalysts. A comprehensive comparison with previously reported catalysts is given in Table 1.

**Table 1:** Comparison of electrocatalytic activity with previous reported catalysts

Catalysts	OP^a^ (V)	*η* ^b^ (V) (at 10 mA cm^−2^)	TS^c^ (mV dec^−1^)	Electrolyte	References
Porous Co_3_O_4_ nanoplates	1.514	0.523	71	0.1 M KOH	[40]
Co_3_O_4_/mildly oxidized MCNT	1.51	0.39	65	0.1 M KOH	[41]
CoO/N-doped crumpled graphene	N. A.	0.34	71	0.1 M KOH	[42]
Au-meso-Co_3_O_4_	1.53	0.44	46	0.1 M KOH	[43]
Hollow Ni–Co oxide nanosponges	1.50	0.36	61	0.1 M KOH	[44]
Rutile RuO_2_	>1.70	141	N. A.	0.1 M KOH	[45]
IrO_2_/C	N. A.	0.37	N. A.	0.1 M KOH	[21]
This work	1.41	0.30	84	0.1 M KOH	–

^a^ OP = onset potential; ^b^ *η* = over-potential at current density of 10 mA cm^−2^; ^c^ TS = Tafel slope

**Fig. 11:**
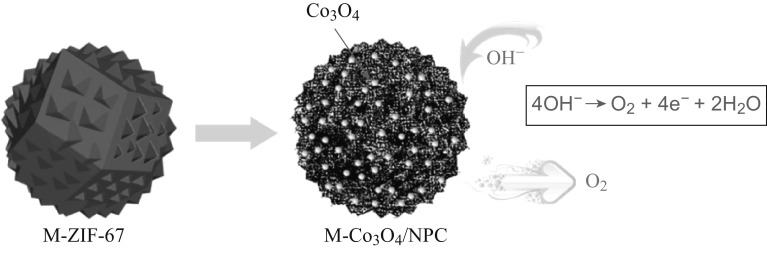
Schematic diagram of the M-Co_3_O_4_/NPC structure

The reason for better electrocatalytic performance of M-Co_3_O_4_/NPC over the other two composites was attributed to its favorable structure (Fig. 11). Firstly, the M-Co_3_O_4_/NPC composite derived from the flower-like ZIF-67 was comprised of the nitrogen-doped carbon scaffold with uniformly attached Co_3_O_4_ nanoparticles. The unique carbon network provided channels for the electrolyte, allowing intimate contact between the electrode and the electrolyte, hence promoting interfacial charge transfer. Besides, good electrical conductivity of the carbon scaffold likely also facilitated the electron transport. Thirdly, a highly disordered structure implied the presence of more active sites, which were key to the improvement in OER activity. Furthermore, the flower-like carbon matrix showed high structural stability, which could firmly support the Co_3_O_4_ nanoparticles and thus improved the stability of the catalyst.

## Conclusion

In summary, a facile method for the preparation of Co_3_O_4_/NPC composites with different morphologies has been proposed, in which Co_3_O_4_ nanoparticles were uniformly embedded in a nitrogen-doped carbon scaffold. By slightly modulating the synthetic route of the ZIF-67 precursors, it was possible to achieve control of their morphology. This facile method provided a new means to prepare MOF-derived electrocatalysts for the OER. Among the three Co_3_O_4_/NPC composites, the M-Co_3_O_4_/NPC derived from the flower-like ZIF-67 displayed superior electrocatalytic activity. The excellent performance of the M-Co_3_O_4_/NPC composite was attributed to its favorable structure. Firstly, the unique carbon network allowed an intimate contact area between the electrode and the electrolyte, thus promoting interfacial charge transfer. Secondly, the highly disordered structure resulted in more active sites, which were determinant to the electrocatalytic activity for OER. Lastly, the flower-like carbon matrix assumed high structural stability, which firmly supported the Co_3_O_4_ nanoparticles, thus improving the stability of the catalyst.

## Electronic supplementary material

Below is the link to the electronic supplementary material.

Supplementary material 1 (PDF 554 kb)

## References

[1] Winter M, Brodd RJ (2005). What are batteries, fuel cells, and supercapacitors?. Chem. Rev..

[2] Chu S, Majumdar A (2012). Opportunities and challenges for a sustainable energy future. Nature.

[3] Zhang P, Zhang JJ, Gong JL (2014). Tantalum-based semiconductors for solar water splitting. Chem. Soc. Rev..

[4] Liang JS, Liu KY, Li SZ, Wang DZ, Ren TQ, Xu XY, Luo Y (2015). Novel flow field with superhydrophobic gas channels prepared by one-step solvent-induced crystallization for micro direct methanol fuel cell. Nano-Micro Lett..

[5] Kim TW, Choi KS (2014). Nanoporous BiVO_4_ photoanodes with dual-layer oxygen evolution catalysts for solar water splitting. Science.

[6] Zhang M, de Respinis M, Frei H (2014). Time-resolved observations of water oxidation intermediates on a cobalt oxide nanoparticle catalyst. Nat. Chem..

[7] Guo K, Li Y, Yang J, Zou ZQ, Xue XZ, Li XM, Yang H (2014). Nanosized Mn–Ru binary oxides as effective bifunctional cathode electrocatalysts for rechargeable LiO_2_ batteries. J. Mater. Chem. A.

[8] Tian GL, Zhao MQ, Yu DS, Kong XY, Huang JQ, Zhang Q, Wei F (2014). Nitrogen-doped graphene/carbon nanotube hybrids: in situ formation on bifunctional catalysts and their superior electrocatalytic activity for oxygen evolution/reduction reaction. Small.

[9] Parent AR, Sakai K (2014). Progress in base-metal water oxidation catalysis. Chemsuschem.

[10] Jin K, Park J, Lee J, Yang KD, Pradhan GK (2014). Hydrated manganese(II) phosphate (Mn-3(PO_4_)(2)center dot 3H(2)O) as a water oxidation catalyst. J. Am. Chem. Soc..

[11] Gong M, Li YG, Wang HL, Liang YY, Wu JZ (2013). An advanced Ni–Fe layered double hydroxide electrocatalyst for water oxidation. J. Am. Chem. Soc..

[12] Subbaraman R, Tripkovic D, Chang KC, Strmcnik D, Paulikas AP (2012). Trends in activity for the water electrolyser reactions on 3d M(Ni Co, Fe, Mn) hydr(oxy)oxide catalysts. Nat. Mater..

[13] Xing M, Kong LB, Liu MC, Liu LY, Kang L, Luo YC (2014). Cobalt vanadate as highly active, stable, noble metal-free oxygen evolution electrocatalyst. J. Mater. Chem. A.

[14] Phihusut D, Ocon JD, Jeong B, Kim JW, Lee JK, Lee J (2014). Gently reduced graphene oxide incorporated into cobalt oxalate rods as bifunctional oxygen electrocatalyst. Electrochim. Acta.

[15] Wee TL, Sherman BD, Gust D, Moore AL, Moore TA, Liu Y, Scaiano JC (2011). Photochemical synthesis of a water oxidation catalyst based on cobalt nanostructures. J. Am. Chem. Soc..

[16] Zhao HX, Zheng Z, Li J, Jia HM, Wong KW, Zhang YD, Lau WM (2013). Substitute of expensive Pt with improved electrocatalytic performance and higher resistance to CO poisoning for methanol oxidation: the case of synergistic Pt-Co_3_O_4_ nanocomposite. Nano-Micro Lett..

[17] Sa YJ, Kwon K, Cheon JY, Kleitz F, Joo SH (2013). Ordered mesoporous Co_3_O_4_ spinels as stable, bifunctional, noble metal-free oxygen electrocatalysts. J. Mater. Chem. A.

[18] Grewe T, Deng XH, Weidenthaler C, Schuth F, Tuysuz H (2013). Design of ordered mesoporous composite materials and their electrocatalytic activities for water oxidation. Chem. Mater..

[19] Leng X, Zeng QC, Wu KH, Gentle IR, Wang DW (2015). Reduction-induced surface amorphization enhances the oxygen evolution activity in Co_3_O_4_. RSC Adv..

[20] Bergmann A, Martinez-Moreno E, Teschner D, Chernev P, Gliech M, de Araujo JF, Reier T, Dau H, Strasser P (2015). Reversible amorphization and the catalytically active state of crystalline Co_3_O_4_ during oxygen evolution. Nat. Commun..

[21] Zhao Y, Nakamura R, Kamiya K, Nakanishi S, Hashimoto K (2013). Nitrogen-doped carbon nanomaterials as non-metal electrocatalysts for water oxidation. Nat. Commun..

[22] Nagaiah TC, Bordoloi A, Sanchez MD, Muhler M, Schuhmann W (2012). Mesoporous nitrogen-rich carbon materials as catalysts for the oxygen reduction reaction in alkaline solution. Chemsuschem.

[23] Gong KP, Du F, Xia ZH, Durstock M, Dai LM (2009). Nitrogen-doped carbon nanotube arrays with high electrocatalytic activity for oxygen reduction. Science.

[24] Liang YY, Li YG, Wang HL, Zhou JG, Wang J, Regier T, Dai HJ (2011). Co_3_O_4_ nanocrystals on graphene as a synergistic catalyst for oxygen reduction reaction. Nat. Mater..

[25] Chen P, Xiao TY, Qian YH, Li SS, Yu SH (2013). A nitrogen-doped graphene/carbon nanotube nanocomposite with synergistically enhanced electrochemical activity. Adv. Mater..

[26] Wei Y, Zhang XY, Luo ZY, Tang D, Chen CX, Zhang T, Xie ZL (2017). Nitrogen-doped carbon nanotube-supported Pd catalyst for improved electrocatalytic performance toward ethanol electrooxidation. Nano-Micro Lett..

[27] Lee JS, Park GS, Kim ST, Liu ML, Cho J (2013). A highly efficient electrocatalyst for the oxygen reduction reaction: N-doped ketjenblack incorporated into Fe/Fe_3_C-functionalized melamine foam. Angew. Chem. Int. Ed..

[28] Silva R, Voiry D, Chhowalla M, Asefa T (2013). Efficient metal-free electrocatalysts for oxygen reduction: polyaniline-derived N- and O-doped mesoporous carbons. J. Am. Chem. Soc..

[29] Zhang JT, Zhao ZH, Xia ZH, Dai LM (2015). A metal-free bifunctional electrocatalyst for oxygen reduction and oxygen evolution reactions. Nat. Nanotechnol..

[30] Wu G, More KL, Johnston CM, Zelenay P (2011). High-performance electrocatalysts for oxygen reduction derived from polyaniline, iron, and cobalt. Science.

[31] Du J, Cheng FY, Wang SW, Zhang TR, Chen J (2014). M(Salen)-derived nitrogen-doped M/C (M = Fe Co, Ni) porous nanocomposites for electrocatalytic oxygen reduction. Sci. Rep..

[32] Chen YQ, Li JT, Yue GH, Luo XT (2017). Novel Ag@nitrogen-doped porous carbon composite with high electrochemical performance as anode materials for lithium-ion batteries. Nano-Micro Lett..

[33] Li C, Chen T, Xu W, Lou X, Pan L, Chen Q, Hu B (2015). Mesoporous nanostructured Co_3_O_4_ derived from MOF template: a high-performance anode material for lithium-ion batteries. J. Mater. Chem. A.

[34] Liu HZ, Xia GL, Zhang RR, Jiang P, Chen JT, Chen QW (2017). MOF-derived RuO_2_/Co_3_O_4_ heterojunctions as highly efficient bifunctional electrocatalysts for HER and OER in alkaline solutions. RSC Adv..

[35] Antony RP, Satpati AK, Bhattacharyya K, Jagatap BN (2016). MOF derived nonstoichiometric NixCo_3−x_O_4−y_ nanocage for superior electrocatalytic oxygen evolution. Adv. Mater. Interfaces.

[36] Ming FW, Liang HF, Shi HH, Xu X, Mei G, Wang ZC (2016). MOF-derived Co-doped nickel selenide/C electrocatalysts supported on Ni foam for overall water splitting. J. Mater. Chem. A.

[37] Millward AR, Yaghi OM (2005). Metal-organic frameworks with exceptionally high capacity for storage of carbon dioxide at room temperature. J. Am. Chem. Soc..

[38] He T, Chen DR, Jiao XL, Wang YL, Duan YZ (2005). Solubility-controlled synthesis of high-quality Co_3_O_4_ nanocrystals. Chem. Mater..

[39] Li XZ, Fang YY, Lin XQ, Tian M, An XC, Fu Y, Li R, Jin J, Ma JT (2015). MOF derived Co_3_O_4_ nanoparticles embedded in N-doped mesoporous carbon layer/MWCNT hybrids: extraordinary bi-functional electrocatalysts for OER and ORR. J. Mater. Chem. A.

[40] Zhou XM, Xia ZM, Tian ZM, Ma YY, Qu YQ (2015). Ultrathin porous Co_3_O_4_ nanoplates as highly efficient oxygen evolution catalysts. J. Mater. Chem. A.

[41] Lu XY, Zhao C (2013). Highly efficient and robust oxygen evolution catalysts achieved by anchoring nanocrystalline cobalt oxides onto mildly oxidized multiwalled carbon nanotubes. J. Mater. Chem. A.

[42] Mao S, Wen ZH, Huang TZ, Hou Y, Chen JH (2014). High-performance bi-functional electrocatalysts of 3D crumpled graphene-cobalt oxide nanohybrids for oxygen reduction and evolution reactions. Energy Environ. Sci..

[43] Lu XY, Ng YH, Zhao C (2014). Gold nanoparticles embedded within mesoporous cobalt oxide enhance electrochemical oxygen evolution. Chemsuschem.

[44] Zhu CZ, Wen D, Leubner S, Oschatz M, Liu W, Holzschuh M, Simon F, Kaskel S, Eychmuller A (2015). Nickel cobalt oxide hollow nanosponges as advanced electrocatalysts for the oxygen evolution reaction. Chem. Commun..

[45] Lee Y, Suntivich J, May KJ, Perry EE, Shao-Horn Y (2012). Synthesis and activities of rutile IrO_2_ and RuO_2_ nanoparticles for oxygen evolution in acid and alkaline solutions. J. Phys. Chem. Lett..

