# Supplementary material for: Novel Co3O4 Nanoparticles/Nitrogen-Doped Carbon Composites with Extraordinary Catalytic Activity for Oxygen Evolution Reaction (OER)
Source: Nanomicro Lett. 2017 Nov 14;10(1):15. doi: 10.1007/s40820-017-0170-4 (PMC6199064; doi:10.1007/s40820-017-0170-4)
Supplement: Supplementary file 1 — Supplementary material 1 (PDF 554 kb) [file 40820_2017_170_MOESM1_ESM.pdf]

Supporting Information for

Novel Co_3O_4 Nanoparticles/Nitrogen-Doped Carbon Composites with Extraordinary Catalytic Activity for Oxygen Evolution Reaction (OER)

Xiaobing Yang[1, 3, †], Juan Chen[2, †], Yuqing Chen[4], Pingjing Feng[4], Huixian Lai[4], Jintang Li[4], Xuetao Luo[4,*]

[1]College of Ecology and Resource Engineering, Wuyi University, Fujian Wuyishan, 354300, China.
[2]Fujian Key Laboratory of Advanced Materials, College of Materials, Xiamen University, Xiamen 361005, People's Republic of China
[3]Fujian Provincial Key Laboratory of Eco-Inductrial Green Technology, Wuyi University, Fujian Wuyishan, 354300, People's Republic of China
[4]Department of Pharmacy, Zhongshan Hospital, Xiamen University, Xiamen 361004, People's Republic of China

†Yuqing Chen and Juan Chen contributed equally to this work.
*Corresponding author. E-mail: xuetao@xmu.edu.cn

Tel: 86-0592-2188503

Figures and Tables

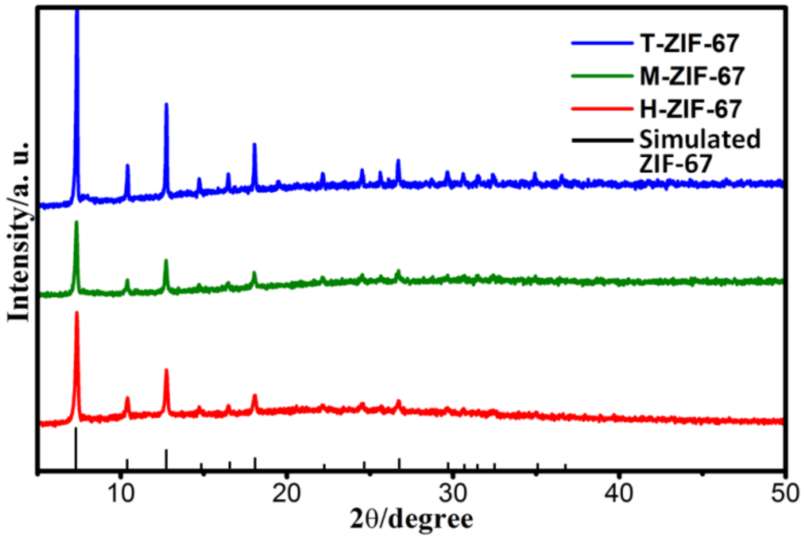

Fig. S1 PXRD patterns of M-ZIF-67, H-ZIF-67, and T-ZIF-67

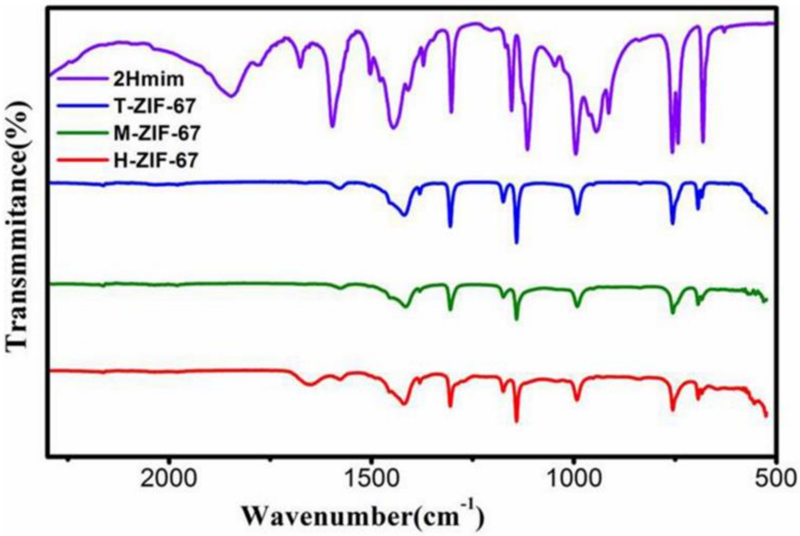

Fig. S2 FTIR spectrum of 2-Hmim, M-ZIF-67, H-ZIF-67, and T-ZIF-67

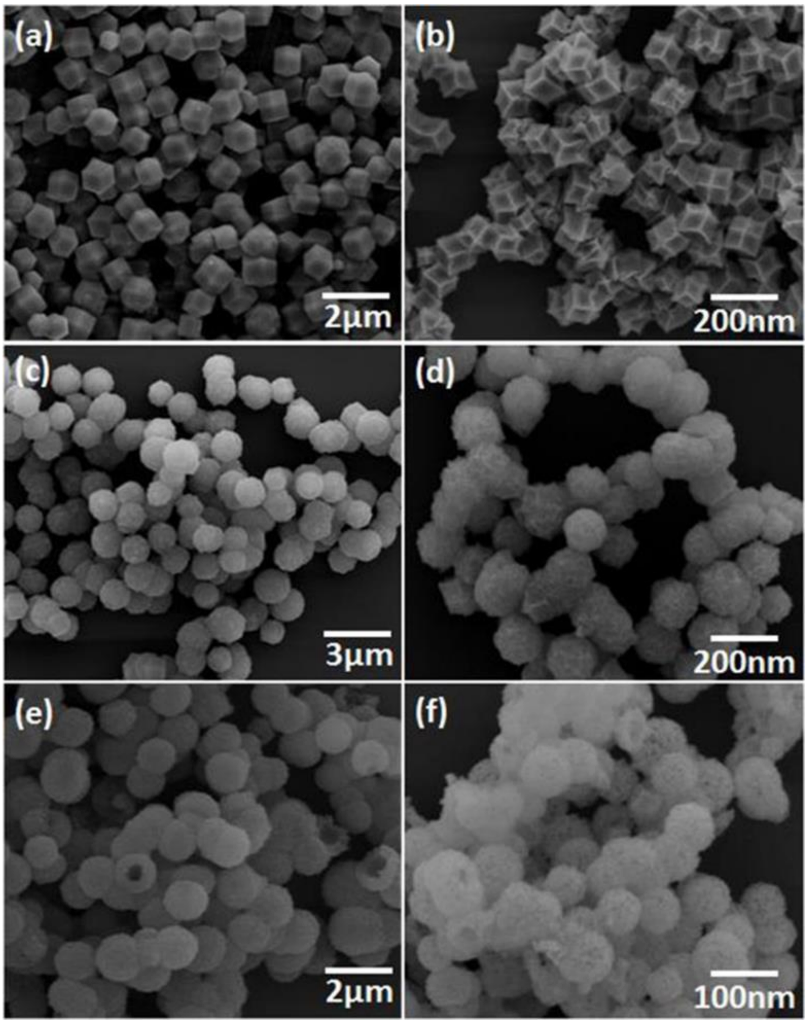

Fig. S3 SEM images of **a** T-ZIF-67, **b** T-Co_3O_4/NPC, **c** M-ZIF-67, **d** M-Co_3O_4/NPC, **e** H-ZIF-67, and **f** H-Co_3O_4/NPC

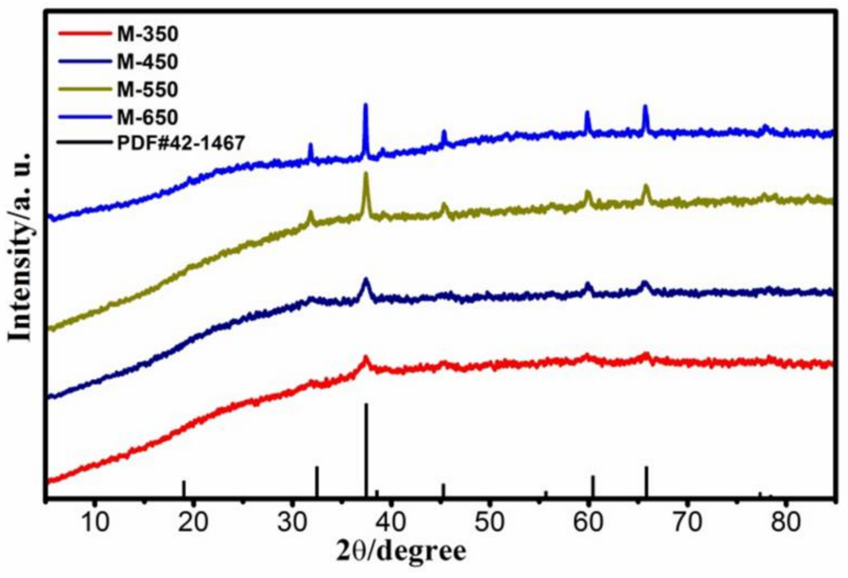

Fig. S4 powder XRD of M-350, M-450, M-550, and M-660

Table S1 elemental contents of Co_3O_4/NPC composites

	Co	C	N	O	Co/O
T-Co_3O_4/NPC	62.43	11.41	0.56	25.60	2.44
M-Co_3O_4/NPC	60.22	15.50	0.8	23.48	2.56
H-Co_3O_4/NPC	54.16	22.33	1.25	22.29	2.43
